# Malware Detection Using RNA Encoding and Convolutional Neural Networks on the Malicious Network Dataset

**DOI:** 10.12688/f1000research.173837.1

**Published:** 2026-02-12

**Authors:** Omar Fitian Rashid, Senan Ali Abd, Humam Al-Shahwani

**Affiliations:** 1 Department of Geology, University of Baghdad, Baghdad, Baghdad Governorate, Iraq; 2 Department of Cybersecurity, College of Information Technology, University of Fallujah, Al-Fallujah, Al Anbar Governorate, Iraq; 3 Department of Computer Science, College of Science, University of Baghdad, Baghdad, Baghdad Governorate, Iraq

**Keywords:** Malware Detection, RNA Encoding, Convolutional Neural Networks, Network Security, Malicious Network Dataset

## Abstract

**Background:**

The detection of malware in network traffic remains a critical cybersecurity challenge. Traditional signature-based intrusion detection demonstrates a high level of familiarity with issues that have been recorded in the database; but show significantly lower effectiveness when it comes to polymorphic or zero-day attacks. Conversely, anomaly-based approaches are also endowed with the ability to detect new incursions, but often have a high false-positive rate.

**Methods:**

This study proposes a combined malware-detection framework which makes use of RNA encoding network-flow attributes alongside Convolutional Neural Network (CNN) classifiers. The framework has three functionalities: a Signature-CNN, which is trained on RNA-encoded representation of known malicious flows; an Anomaly-CNN, which is developed to distinguish between benign and malicious traffic without any signature prior knowledge; and a Hybrid-CNN, which combines both paradigms in a two-stage detection pipeline.

**Results:**

Experiments conducted on the Malicious Network Dataset show that the Signature-CNN achieves 91% accuracy with strong precision on known threats, the Anomaly-CNN achieves 93% detection rate on unknown malware, and the Hybrid-CNN achieves the best overall performance with 95% detection rate and 94.5% F1 score.

**Conclusions:**

The results demonstrate that RNA encoding combined with CNN classifiers offers a robust and scalable solution for malware detection in networked environments.

## 1. Introduction

The exponential growth of networked systems proliferation has led to an equivalent increase in advanced malware attacks. Intrusion detection systems (IDS) continue to play a central role in the protection of digital infrastructure; however, modern practices are characterized by severe limitations. ^1^ Signature-based IDS rely on a set of pre-defined rules or patterns, and these systems are effective only against previously identified threats. Anomaly-based IDS attempt to detect deviations from normal behavior, and has the ability to detect zero-day attacks, but often generate a high false positive. To address these limitations, a hybrid architecture has arisen, combining the benefits of each of the system. ^2, 3^

There have been recent studies into how deep-learning architectures, especially convolutional neural networks (CNNs) and recurrent neural networks (RNNs), can be applied to the problems of IDS, with promising results. A novel intrusion detection method based on learning framework is proposed, ^4^ where the proposed method is done by using dual parallel CNN pipelines to independently address the network and radar features. A new intrusion detection model is suggested by, ^5^ where this model combines CNN and Random Forest. The CNN is utilized to extract the feature, and the Random Forest is used for classification. An IDS is proposed by combining an innovative hybrid Autoencoder with an enhanced LSTM-CNN architecture, ^6^ where the proposed method can enhance the detection capabilities more quickly and efficiently. Kaissar et al. ^7^ investigates the optimization of hyperparameters in CNN to enhance the NIDS performance, where Grid Search, Genetic Algorithm, Particle Swarm Optimization, and Grey Wolf Optimization algorithms are used for this purpose. Alrayes et al. ^8^ suggested a novel IDS by combining channel attention and CNN, where the suggested method has exceptional accuracy when applied it to NSL-KDD dataset. A new IDS model is built based on CNN and knowledge distillation, ^9^ this model using two-dimensional Fourier transform for converting the grayscale images to the frequency domain, and this led to enhanced the similarity between neighboring pixels to address data effectively. Ban et al. ^10^ suggested an enhanced deep-learning model for IDS in IoT environment, where the suggested model is depending on CNN as the backbone network in the constructed model. A hybrid deep learning IDS is proposed by ^11^ based on CNN and bidirectional long short-term memory neural networks, where the proposed system is enhanced the model’s ability to detect patterns in both minority and majority classes. Altunay and Albayrak ^12^ developed IDS in the IIoT networks, where the suggest system is done by using three different deep learning architectures, which are CNN, Long Short-Term Memory (LSTM), and the combination of these two methods.

Parallel Encodings Biologically inspired encodings, like mappings to DNA and RNA sequences, have been proposed to convert heterogeneous data to symbolic strings. ^13, 14^ Nevertheless, there is limited evidence in the extant literature of the integration of RNA encodings with CNN classifiers in the entire range of detection paradigms: signature, anomaly, and hybrid. The current paper seals this gap by suggesting a CNN-based model which incorporates these complementary detection schemes.

## 2. Methodology

**Figure 1.**
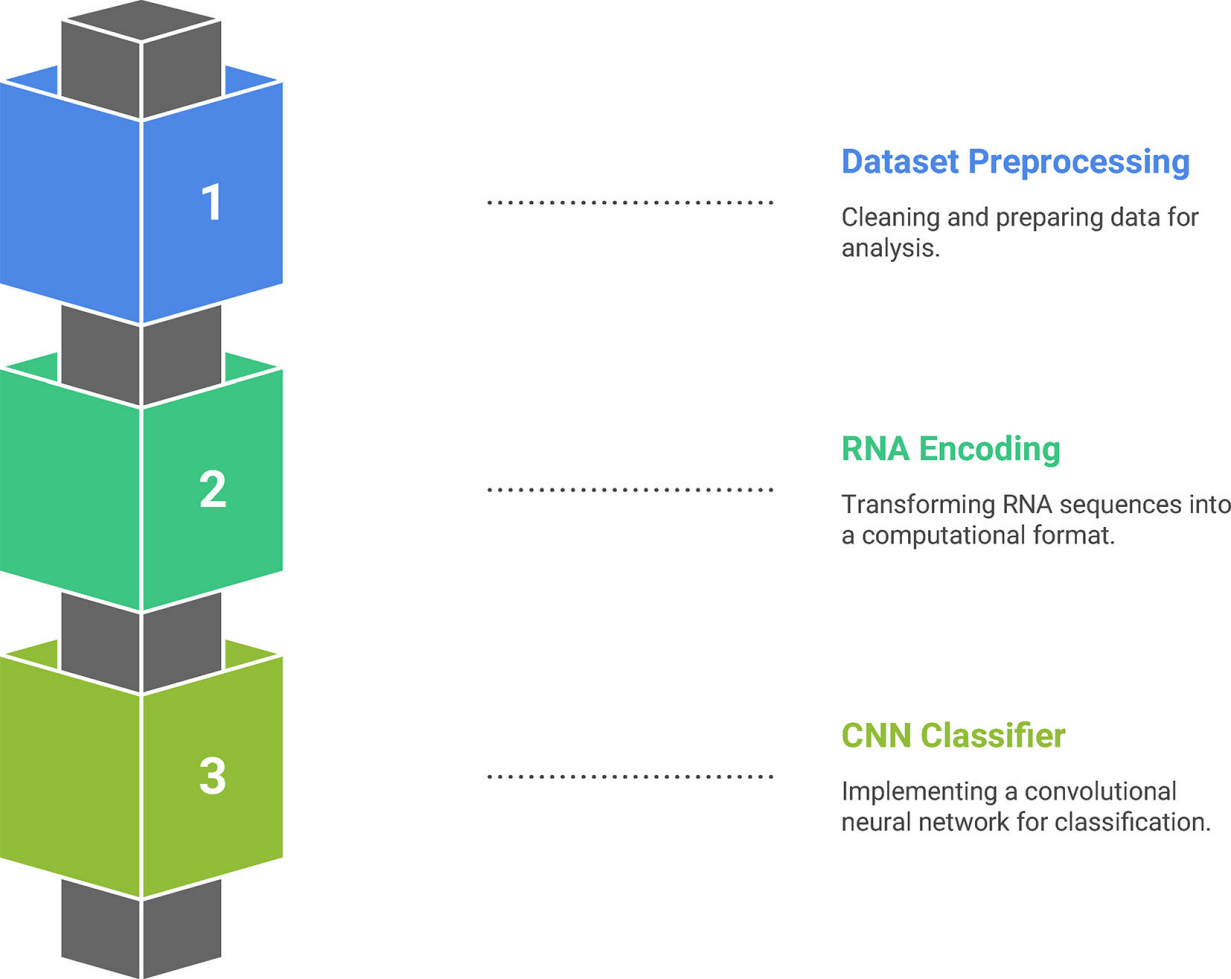
Flow chart of the suggested malware detection system with RNA encoding and CNN classification pipeline.

The proposed malware detection system is built by combining of RNA-inspired encoding of the network traffic characteristics and the convolutional neural network (CNN) classification. Unlike traditional intrusion detection system models that treat signature-based and anomaly-based detection methodologies as dissimilar entities, the current system integrates both of them in a single deep learning pipeline. In this pipeline, CNN models that are trained on sequences coded using the RNA-inspired methodology concurrently address signature-based, anomaly-based, and hybrid detection. The steps of the proposed system are shown in Figure 1.

### 2.1 Dataset description

The Malicious Network Dataset is a new dataset that was collected using honeypots deployed with the Honeytrap agent. The dataset consists of 9 features that represent various aspects of network traffic, including both structural and payload data. ^15^ These features are shown in Table 1 as follows:

**Table 1.** The malicious network dataset values and its descriptions. ^15^

Feature	Column	Possible values
**1**	**Protocol**	TCP, IP
**2**	**remote_ip**	Too many unique values (10,951). Example: ['35.203.211.180', '180.93.172.180', '81.17.19.66', '0.0.0.0', '94.23.145.155']
**3**	**remote_port**	Numeric range: 0 – 65535 (unique=28,920)
**4**	**local_ip**	Too many unique values (4,030). Example: ['165.227.180.71', '0.0.0.0', '192.168.202.139', '157.240.11.61', '192.168.11.143']
**5**	**local_port**	Numeric range: 0 – 65535 (unique=11,985)
**6**	**md5_hash**	Too many unique values (13,732). Example: ['1fb4aeaab94ca27d2e5dfaa47e11a6fb', 'd41d8cd98f00b204e9800998ecf8427e', '19b893b938ace1defe7d090e510f0618', “, '59b490c4ab003464ca03428b3fc63222']
**7**	**sha512_hash**	Too many unique values (13,732). Example: ['a6d4f36a2a8d5b5ea7e6afe91d4a80e7a9ae2129ebf4791a4d74b7ea003420c2…', 'cf83e1357eefb8bdf1542850d66d8007…', 'f060846cbf02e31706d5d0fe781d7007…', “, '922450f93e933de877934cee97339ae6…']
**8**	**Length**	Numeric range: 0 – 1448 (unique = 1,038)
**9**	**data_hex**	Too many unique values (13,732). Example: ['16030100ca010000c60303918984ed51d5c0b8d4cfad730a16a4efb24b004062e21…', “, '50100', '0', '474554202f20485454502f312e310d0a486f7374…']
**10**	**Class**	'malicious' or 'benign'.

### 2.2 RNA encoding of network features

Network flows in the Malicious Network Dataset comprise heterogeneous attributes, such as protocol types, port numbers, cryptographic hashes, packet lengths, and payloads. Such attributes are of different scales and representation thus complicating direct modeling. In this spirit we introduce a biologically inspired RNA encoding scheme where each element is coded to a fixed set of codons. Where the mapping rules as follow:

○ Remote ip and local ip attributes were eliminated, because a malware detection model must identify malicious signatures regardless of the source and destination IP addresses, and these ips do not provide meaningful behavioral indicators of malware.
○ Protocol identifiers (e.g., TCP, IP) are assigned codons such as TCP → G, IP → U.
○ Numerical fields (ports, lengths) are separated into digits, each mapped to a codon, where each digit is represented by two RNA characters, e.g., 0 → CG, 1 → AC, and so on.
○ Hexadecimal payload values are mapped similarly, where each character is represented by two RNA characters, e.g., a → AU, b → UU, and so on.
○ The hash values, containing both MD5 and SHA512 are divided into single characters and coded into codons, thus, making sure that each unique character is represented by a deterministic codon.
○ For each flow, codon sequences from all fields are concatenated in the following order: [protocol] → [remote port] → [local port] → [MD5] → [SHA512] → [length] → [payload].
○ The built RNA encoding for all possible malicious network dataset records values is shown in Table 2.

**Table 2.** RNA encoding for malicious network dataset records values.

Value	RNA encoding
TCP	G
IP	U
0	CG
1	AC
2	GG
3	UA
4	CC
5	GA
6	UC
7	AA
8	GU
9	UG
a	AU
b	UU
c	CA
d	AG
e	CU
f	GC

This mapping transforms a wide range of categorical and string features into structured RNA codon sequences, thus making training of convolutional neural networks on homogeneous sequential inputs possible.

### 2.3 CNN architecture

**Figure 2.**
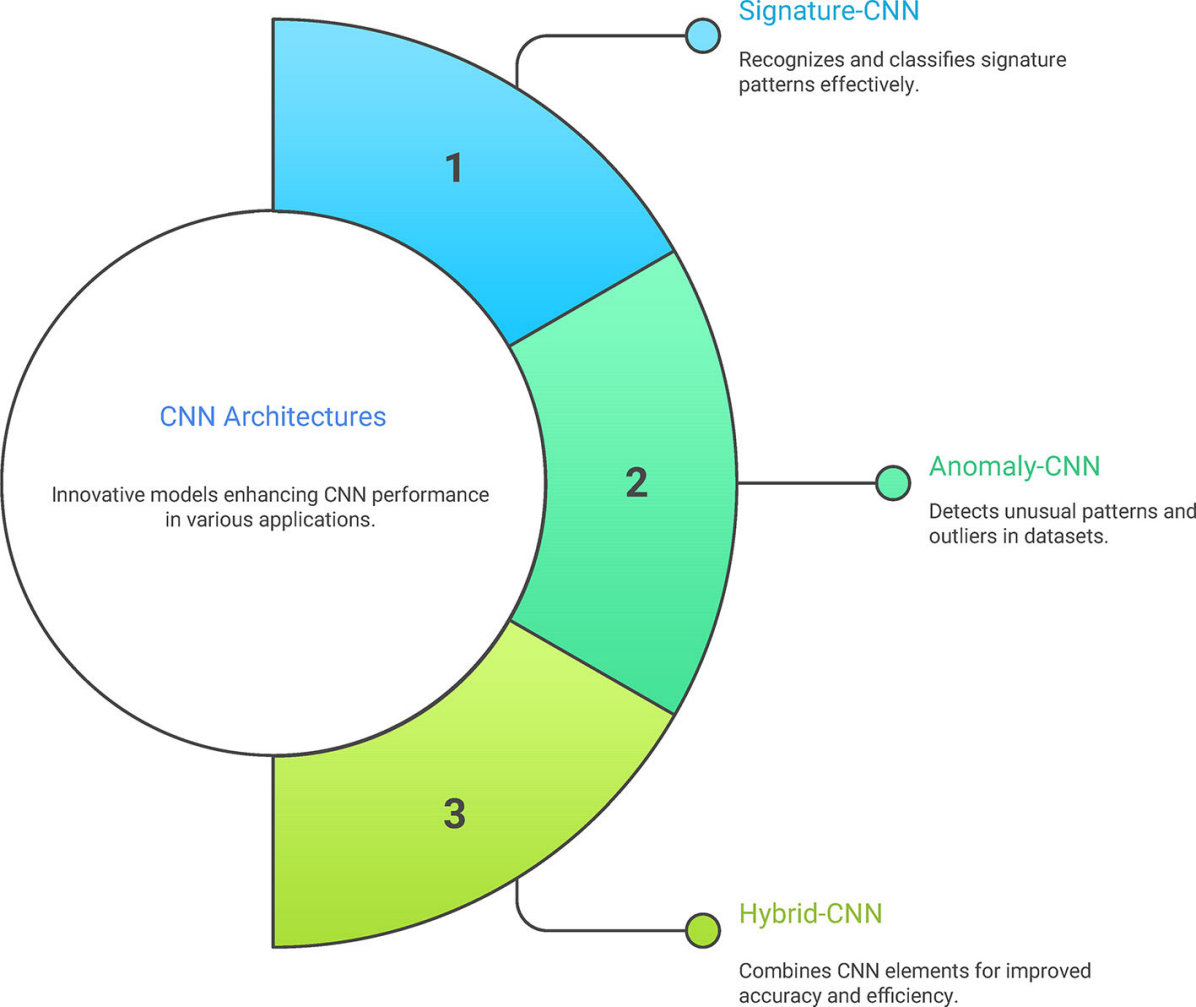
The three CNN models (Signature, Anomaly, and Hybrid).

The coded messages are fed into a Convolutional Neural Network (CNN) that picks up discriminative features at a variety of levels of abstraction as follow:

o **Embedding:** The codons are first mapped to dense vectors of dimension, d = 32. This embedding is learnt alongside the classifier, thus, encoding similarities between codons.
o **Convolutional:** A number of one-dimensional convolutional layers are used, the size of which varies between 5 and 7. These filters identify a local pattern in the codon sequences e.g., repeated sequences which can be an indication of malicious activity. An example would be to have a convolution filter that is trained to identify the codon sequence of known back door ports.
o **Pooling:** The feature maps are down sampled through max-pooling, and the most conspicuous features are retained, with a lower computational cost.
o **Global Average Pooling:** In order to generalize over a wide range of lengths of variable sequences and reduce overfitting, global average pooling is done to aggregate the feature maps into fixed-size vectors.
o **Dense Layers:** The learned features are combined in fully connected layers (64 units) that use ReLU activation. To inhibit overfitting, dropout regularization is used, with a temporary activation of neurons in the course of training, i.e. p = 0.5.
o **Output Layer:** One sigmoid neuron generates a probability score which can mark a sample to be benign or malicious.
o The CNN architecture is applied for three different methods, and these methods are shown in Figure 2.

In Signature-CNN, the Signature- CNN replaces traditional rule-based signature matching with a convolutional neural network, which is trained on representations of known patterns of malicious activity encoded by RNA. Instead of searching manually through the collection of byte sequences or hash values, the network is trained to identify codon-level motifs which are indicative of malicious flows. While the Anomaly- CNN is trained to identify deviation with the normal network behavior using sequences encoded by RNA. It is not based on predefined attack patterns as compared to the signature model. Finally, the Hybrid-CNN combines the two methods in two-staged pipeline, allowing the Signature-CNN to combine the precision of Anomaly-CNN with the generalization capability. The comparison between the three CNN models is clarified in Table 3.

**Table 3.** Comparison between different CNN models.

Model	Input	Objective	Strengths	Weaknesses
Signature-CNN	RNA-coded sequences of known malicious and benign flows	Detect known attack patterns using learned codon-level motifs	High precision and low FAR	Cannot detect zero-day threats
Anomaly-CNN	RNA-coded mixture of benign and malicious flows	Identify deviations from normal codon distributions	Detects novel and polymorphic malware	Higher false-positive rate
Hybrid-CNN	Two-stage input: Signature-filtered and residual flows	Combine the benefits of both precision and generalization	Best overall accuracy and balance	Slightly higher computational cost

Where the sequence handling all the RNA sequences were truncated or zero padded to a constant length of 2,048 codons to make the input equal. Also, the architectural consistency for the CNN models of the three models have the same architecture (three Conv1D layers with 64-128-256 filters, kernel = 5, ReLU activation, and dropout = 0.5). It is only in training objectives that there is a difference. Finally, the dataset was stratified 70/30 and sampled to maintain the malicious/benign ratio (50/50). These explanations guarantee the maximum reproducibility of the experiment.

## 3. Results and discussion

The performance of the proposed CNN-based malware detection models is evaluated based on several standard classification metrics were employed, where these metrics are defined and calculated as follow:

○ Accuracy: Is the ratio of correctly labeled flows to the total number of flows, and it calculate based on the following equation:
$$\text{Accuracy}=\frac{\mathrm{TP}+\mathrm{TN}}{\mathrm{TP}+\mathrm{TN}+\mathrm{FP}+\mathrm{FN}}$$

○ Detection Rate (Recall): This rate also known as the True Positive Rate (TPR) is a metric that measures the rate of true malicious flows that are detected, and it calculate based on the following equation:
$$\text{Detection Rate}\;\left(\text{Recall}\right)=\frac{\mathrm{TP}}{\mathrm{TP}+\mathrm{FN}}$$

○ Precision: Refers to the ratio of the flows that are predicted to be malicious which actually are malicious, and it calculate based on the following equation:
$$\text{Precision}=\frac{\mathrm{TP}}{\mathrm{TP}+\mathrm{FP}}$$

○ F1 Score: Is the harmonic mean of Precision and Recall that provides a balanced assessment when there is a tradeoff between the two measures, and it calculate based on the following equation:
$$F1\;\text{Score}=2\times \frac{\text{Precision}\times \text{Recall}}{\text{Precision}+\text{Recall}}$$

○ False Positive Rate (FPR): This value is used to estimate the ratio of false positive results of disproving benign flows, and it calculate based on the following equation:
$$\mathrm{FPR}=\frac{\mathrm{FP}}{\mathrm{FP}+\mathrm{TN}}$$

The Malicious Network Dataset is used for evaluation the proposed method, where each method (signature, anomaly, or hybrid) is starting by preprocessing the used dataset by removing IP fields, then RNA encoding is applied to all remaining features. Then divided the dataset into training and testing, the training is equal to 70%, while the testing used the rest 30% from the whole dataset. The achieved results for the first method (signature-CNN) are shown in Table 4. The Signature-CNN was very accurate and reported a low false-positive rate, thus, justifying its accuracy in identifying known malware.

**Table 4.** The performance of the Signature-CNN model.

Metric	Result
Accuracy	0.915
Detection Rate	0.89
Precision	0.92
F1 Score	0.90
FPR	0.05

While the obtained results based on the second method (anomaly-CNN) are shown in Table 5. The Anomaly-CNN was able to achieve higher rate of detection, which revealed that zero-day threats can be spotted with a slight increase in false positives .

**Table 5.** The performance of the Anomaly-CNN model.

Metric	Result
Accuracy	0.93
Detection Rate	0.93
Precision	0.91
F1 Score	0.92
FPR	0.07

On other hand, when utilized the third method (hybrid-CNN), the achieved results are shown in Table 6. Hybrid-CNN delivered the best trade-off, achieving the best detection rate and F1 score whilst reducing false positives at the same time.

**Table 6.** The performance of the Hybrid-CNN model.

Metric	Result
Accuracy	0.95
Detection Rate	0.95
Precision	0.94
F1 Score	0.945
FPR	0.03

**Figure 3.**
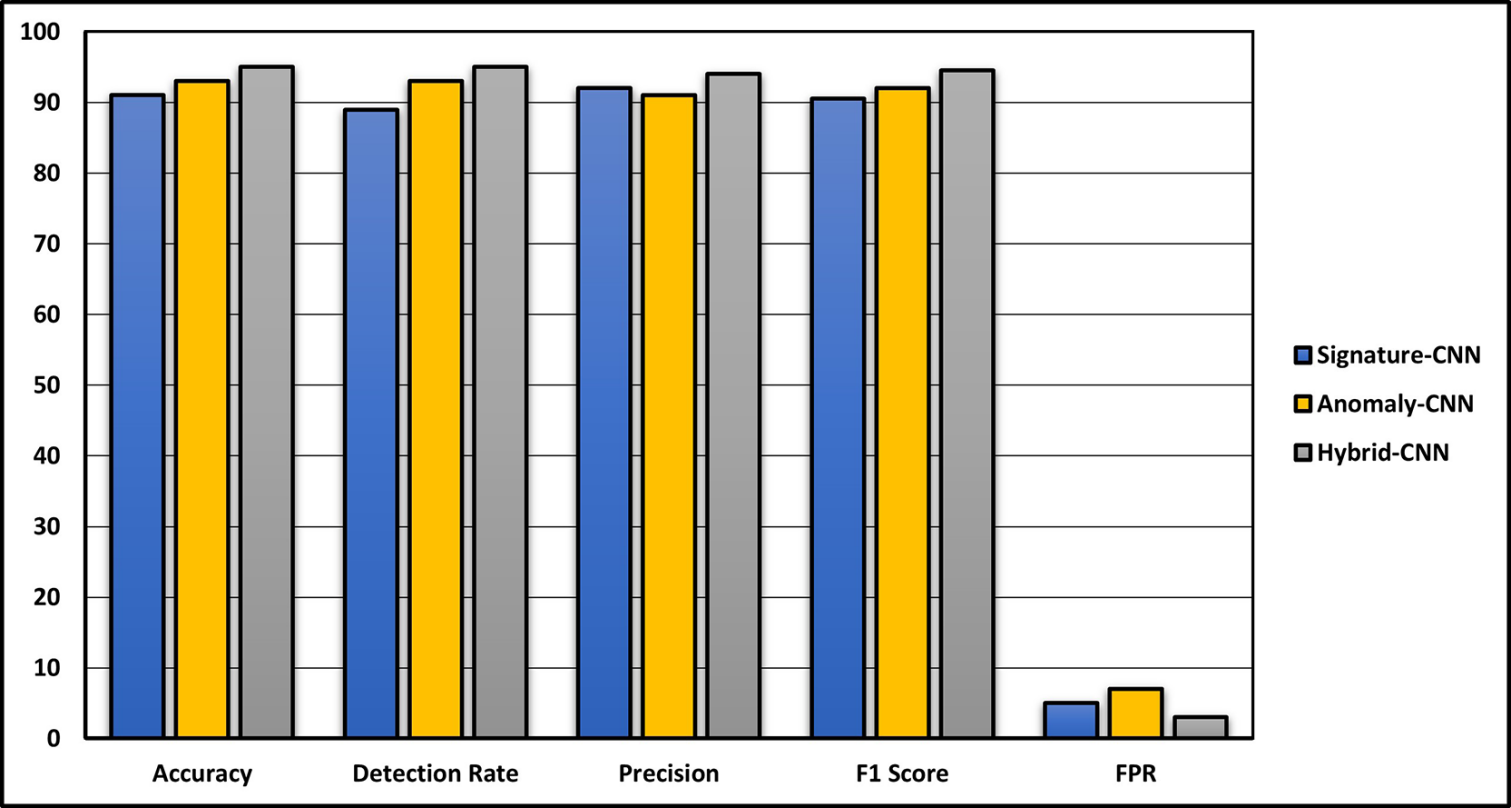
Comparison of Signature-CNN, Anomaly-CNN and Hybrid-CNN on the malicious network dataset.

Finally, the comparison between the performance of all models (signature, anomaly, and hybrid) are shown in Table 7 and Figure 3.

**Table 7.** Performance of CNN models.

Method	Accuracy	Detection rate	Precision	F1 score	FPR
Signature-CNN	0.91	0.89	0.92	0.905	0.05
Anomaly-CNN	0.93	0.93	0.91	0.92	0.07
Hybrid-CNN	0.95	0.95	0.94	0.945	0.03

As shown in Table 7, the achieved results based on hybrid-CNN are achieved the best results over the two others methods, where the obtained accuracy, detection rate, precision, F1 score, and FPR are equal to 95%, 95%, 94%, 94.5%, and 3% respectively. The Hybrid-CNN may be explained by the possibility of making decisions in two stages. The first stage filters familiar malicious patterns and the latter extrapolates to unknown codon patterns. This is a combination that reduces the antagonism of sensitivity and specificity. As compared to the Anomaly- CNN, however, it has a slightly higher recall with more false positives since it does not identify exact patterns but only the deviations. The Signature-CNN is also accurate to known dangers but does not have generalization and this is the reason behind its slightly lower recall.

The proposed method achieved results are compared with classical machine learning models (Random Forest, and XGBoost) and deep models (RNN, CNN-BiLSTM and AE-LSTM) and this comparison is shown in Table 8.

**Table 8.** Comparison of proposed models with existing baselines.

Model	Accuracy	Detection rate	Precision	F1 score	FPR
Random Forest	0.87	0.84	0.86	0.85	0.09
XGBoost	0.89	0.87	0.88	0.875	0.08
RNN	0.91	0.89	0.90	0.895	0.07
LSTM	0.92	0.90	0.91	0.905	0.06
CNN-BiLSTM (Wang et al., 2024)	0.93	0.92	0.92	0.92	0.05
Hybrid AE-LSTM (Xue et al., 2025)	0.94	0.93	0.93	0.93	0.04
**Hybrid-CNN (Proposed)**	**0.95**	**0.95**	**0.94**	**0.945**	**0.03**

As shown in Table 8, The Hybrid-CNN achieved better results than the traditional and deep learning methods. Where the proposed method achieved the highest accuracy, detection rate, precision, and F1-score, where these results are equal to 95%, 95%, 94%, and 94.5% respectively. Also, the obtained FPR results are the lowest and equal to 3%. This has been enhanced by the fact that it has RNA encoding that maintains semantical links between traffic features and increases the pattern recognition capability of the CNN.

### 3.1 Computational efficiency

The experiments were being carried out on the NVIDIA RTX-4090 graphics card with 24 GB VRAM. The Signatures CNN took 1.8 hours for training, Anomaly CNN 2.3 hours and the Hybrid CNN 2.7 hours to training. The mean inference latency (per flow) was 2.1 ms, 2.4 ms and 3.0 ms respectively. Despite the fact that Hybrid-CNN is the most expensive in terms of computation since it involves two stages of evaluation, it is still capable of deployment in near-real-time and takes much less time than recurrent models, including LSTM and AE-LSTM.

## 4. Conclusion

This work proposed an integrated malware detector model based on CNN, which was built on RNA encoding and implemented on Malicious Network Dataset. Restructuring signature, anomaly, and hybrid detection as CNN-based paradigms, the system achieves strong performance across all detection modes. The Hybrid-CNN achieved the best results, having 95% of detection, and the same time, minimized false-positive risks. For future work, the optimization of RNA encoding to stream traffic to allow real-time deployment, lightweight inference latency exploring lightweight architecture like Transformers and Graph Neural Networks, and scaling experiments to larger and more diverse network datasets. Moreover, it will be investigated in association with federated learning models in order to improve data privacy and improve the model generalization across network settings.

## Data Availability

Repository name: Malicious Network Dataset. Zenodo. https://doi.org/10.5281/zenodo.15453468 ^15^ This study uses a publicly available dataset that was originally published by Saadoon and Behadili (2024). The authors did not generate the dataset themselves. The repository contains all underlying data required to reproduce the results reported in this article, including raw network flow records labeled as benign or malicious and all variables used in the experiments (protocol type, port numbers, hash values, payload length, encoded payload data, and class labels). The dataset is openly accessible and released under an open license permitting reuse, with no embargo or access restrictions. Data are available under the terms of the Creative Commons Attribution 4.0 International license (CC-BY 4.0).
