# Supplementary material for: Malware Detection Using RNA Encoding and Convolutional Neural Networks on the Malicious Network Dataset
Source: F1000Res. 2026 May 5;15:241. Originally published 2026 Feb 12. [Version 2] doi: 10.12688/f1000research.173837.2 (PMC13287978; doi:10.12688/f1000research.173837.2)
Supplement: Peer review history

# Reviewer report: Reviewer response for version 2

**DOI:** 10.5256/f1000research.199234.r483152

**Published:** 2026-05-26

**Authors:** Atif Raza Zaidi

**Affiliations:** 1 TIMES University, Multan, Pakistan

The manuscript presents an interesting and relevant study on malware detection using RNA-inspired encoding with CNN-based models. The topic is relevant to network security and malware detection. The proposed approach is interesting, and the reported results are encouraging. However, the following suggestions are offered to further improve technical validation of the work.

 1. The RNA-inspired encoding is a central contribution of the paper. Therefore, an ablation experiment comparing CNN with RNA encoding and CNN without RNA encoding would help show the direct impact of the proposed encoding scheme.

 2. The architecture description mentions a single sigmoid output neuron, while the training description refers to categorical cross-entropy loss. The authors should clarify the exact loss function used and ensure that it is consistent with the output layer configuration.

 3. The manuscript mentions that the dataset was sampled using a 50/50 malicious-benign ratio. However, the exact number of benign and malicious samples should be clearly reported.

 4. The results would be more transparent if confusion matrices were added for Signature-CNN, Anomaly-CNN, and Hybrid-CNN. This would help readers directly observe TP, TN, FP, and FN values behind the reported metrics.

 5. The authors mention that Python and deep learning libraries were used, but the specific tools and library versions are not clearly provided. It would be useful to mention details such as TensorFlow.

 6. Although the manuscript has improved, a final proofreading pass is still recommended to address minor grammatical and stylistic issues. For example, some phrases such as “built by combining of,” and “Where the mapping rules as follow” may be revised for smoother academic readability.

 7. The paper already mentions training settings such as epochs, batch size, optimizer, learning rate, dropout, and early stopping. As an optional improvement, the authors may also consider adding training and validation accuracy/loss curves to show model convergence and overfitting behavior.

Is the work clearly and accurately presented and does it cite the current literature?

Partly

If applicable, is the statistical analysis and its interpretation appropriate?

Partly

Are all the source data underlying the results available to ensure full reproducibility?

Yes

Is the study design appropriate and is the work technically sound?

Yes

Are the conclusions drawn adequately supported by the results?

Partly

Are sufficient details of methods and analysis provided to allow replication by others?

Partly

Reviewer Expertise:

Cybersecurity, Android malware detection, machine learning, and deep learning

I confirm that I have read this submission and believe that I have an appropriate level of expertise to confirm that it is of an acceptable scientific standard, however I have significant reservations, as outlined above.

# Response

**Published:** 2026-05-26

**Authors:** Omar Fitian Rashid

**Reviewer Comments**

**Comment 1:** **The RNA-inspired encoding is a central contribution of the paper. Therefore, an ablation experiment comparing CNN with RNA encoding and CNN without RNA encoding would help show the direct impact of the proposed encoding scheme.**

**Response: **Thank you for this valuable suggestion. An ablation study comparing the CNN model with and without RNA encoding has now been added to better demonstrate the contribution of the proposed RNA-inspired encoding scheme. The additional experiment confirms that RNA encoding improves feature representation and enhances malware detection performance across all evaluation metrics.

**Comment 2:** **The architecture description mentions a single sigmoid output neuron, while the training description refers to categorical cross-entropy loss. The authors should clarify the exact loss function used and ensure that it is consistent with the output layer configuration.**

**Response:** Thank you for identifying this inconsistency. The manuscript has been revised to clarify that the models use a single sigmoid output neuron with binary cross-entropy loss because the task is binary malware classification.

**Comment 3:** **The manuscript mentions that the dataset was sampled using a 50/50 malicious-benign ratio. However, the exact number of benign and malicious samples should be clearly reported.**

**Response:** Thank you for this observation. The manuscript has been revised to explicitly report the number of benign and malicious samples used in the experiments.

**Comment 4:** **The results would be more transparent if confusion matrices were added for Signature-CNN, Anomaly-CNN, and Hybrid-CNN. This would help readers directly observe TP, TN, FP, and FN values behind the reported metrics.**

**Response: **Thank you for this constructive recommendation. Confusion matrices for Signature-CNN, Anomaly-CNN, and Hybrid-CNN have now been added to provide a clearer representation of TP, TN, FP, and FN values.

**Comment 5:** **The authors mention that Python and deep learning libraries were used, but the specific tools and library versions are not clearly provided. It would be useful to mention details such as TensorFlow.**

**Response: **Thank you for the helpful suggestion. The implementation details have now been expanded to include the software environment and deep learning libraries used in the experiments.

**Comment 6:** **Although the manuscript has improved, a final proofreading pass is still recommended to address minor grammatical and stylistic issues. For example, some phrases such as “built by combining of,” and “Where the mapping rules as follow” may be revised for smoother academic readability.**

**Response: **Thank you for the careful review. The manuscript has undergone an additional proofreading pass to improve grammatical accuracy, sentence clarity, and academic readability.

**Comment 7:** **The paper already mentions training settings such as epochs, batch size, optimizer, learning rate, dropout, and early stopping. As an optional improvement, the authors may also consider adding training and validation accuracy/loss curves to show model convergence and overfitting behavior.**

**Response: **Thank you for the careful review. The manuscript has undergone an additional proofreading pass to improve grammatical accuracy, sentence clarity, and academic readability.

# Reviewer report: Reviewer response for version 1

**DOI:** 10.5256/f1000research.191689.r474768

**Published:** 2026-04-21

**Authors:** Mohammed Subhi

**Affiliations:** 1 Directorate of Private University Education,, Baghdad, 10011,, Iraq; 2 Department of Cybersecurity/ Directorate of Information Technology, Iraqi Ministry of Higher Education and Scientific Research, Baghdad, Baghdad, Iraq

The manuscript presents an interesting and timely study on malware detection using RNA encoding combined with CNN architectures. The idea of integrating biologically inspired encoding with deep learning models is promising and shows potential for improving detection performance. However, several issues should be addressed to enhance the clarity, rigor, and overall quality of the manuscript.

 1.    The abstract is generally well-structured; however, it would benefit from including additional details such as the dataset size and key aspects of the experimental setup to improve completeness. 

 2.    The introduction provides a relevant background, but the manuscript requires careful language editing to correct grammatical issues and improve readability. 

 3.    The related work section is comprehensive; however, it would be strengthened by adding a more critical analysis that clearly identifies the limitations of existing approaches and positions the proposed method accordingly. 

 4.    The description of the CNN architecture is clear, but important training details such as optimizer type, learning rate, batch size, and number of epochs are missing and should be provided. 

 5.    The discussion section would benefit from deeper insights into why RNA encoding enhances CNN performance, particularly from a feature representation perspective. 

 6.    The conclusion is appropriate, but the future work section could be made more specific by outlining concrete and actionable research directions.

Is the work clearly and accurately presented and does it cite the current literature?

Partly

If applicable, is the statistical analysis and its interpretation appropriate?

Partly

Are all the source data underlying the results available to ensure full reproducibility?

No source data required

Is the study design appropriate and is the work technically sound?

Yes

Are the conclusions drawn adequately supported by the results?

Partly

Are sufficient details of methods and analysis provided to allow replication by others?

Partly

Reviewer Expertise:

Machine leaarning, artificial intelligience, Iot applications, and AI in security applications

I confirm that I have read this submission and believe that I have an appropriate level of expertise to confirm that it is of an acceptable scientific standard, however I have significant reservations, as outlined above.

# Response

**Published:** 2026-04-21

**Authors:** Omar Fitian Rashid

**Reviewer Comments**

**Comment 1:**

 The abstract is generally well-structured; however, it would benefit from including additional details such as the dataset size and key aspects of the experimental setup to improve completeness.

**Response:**

 Thank you for this valuable suggestion. The abstract has been revised to include the dataset size and key elements of the experimental setup, including the type of data used and evaluation protocol.

**Comment 2:**

 The introduction provides a relevant background, but the manuscript requires careful language editing to correct grammatical issues and improve readability.

**Response:**

 We appreciate this observation. The manuscript has undergone thorough language editing to improve grammar, sentence structure, and overall readability.

**Comment 3:**

 The related work section is comprehensive; however, it would be strengthened by adding a more critical analysis that clearly identifies the limitations of existing approaches and positions the proposed method accordingly.

**Response:**

 Thank you for this insightful comment. A critical analysis has been incorporated to highlight the limitations of existing methods and to clearly position the novelty and advantages of the proposed approach.

**Comment 4:**

 The description of the CNN architecture is clear, but important training details such as optimizer type, learning rate, batch size, and number of epochs are missing and should be provided.

**Response:**

 We appreciate this important suggestion. The training configuration details have been added to ensure reproducibility of the proposed method.

**Comment 5:**

 The discussion section would benefit from deeper insights into why RNA encoding enhances CNN performance, particularly from a feature representation perspective.

**Response:**

 Thank you for this constructive feedback. The discussion has been expanded to provide deeper insights into how RNA encoding improves feature representation and contributes to enhanced CNN performance.

**Comment 6:**

 The conclusion is appropriate, but the future work section could be made more specific by outlining concrete and actionable research directions.

**Response:**

 We appreciate this suggestion. The future work section has been revised to include specific and actionable research directions.
